# Enhancing biliary structure identification using percutaneous cholecystostomy drain delivery of indocyanine green: a glowing two case review

**DOI:** 10.1093/jscr/rjae275

**Published:** 2024-05-02

**Authors:** Peter Alexander, Vincent Marcucci, Patricia Torres, Jillian Cassidy, Seth Kipnis, Dena Arumugam

**Affiliations:** Department of Surgery, St. George’s University, School of Medicine, St. George’sGrenada; Department of Surgery, Jersey Shore University Medical Center, 1945 New Jersey 33, Neptune, NJ, United States; Department of Surgery, Jersey Shore University Medical Center, 1945 New Jersey 33, Neptune, NJ, United States; Department of Surgery, Jersey Shore University Medical Center, 1945 New Jersey 33, Neptune, NJ, United States; Department of Surgery, St. George’s University, School of Medicine, St. George’sGrenada; Department of Surgery, Jersey Shore University Medical Center, 1945 New Jersey 33, Neptune, NJ, United States; Department of Surgery, St. George’s University, School of Medicine, St. George’sGrenada; Department of Surgery, Jersey Shore University Medical Center, 1945 New Jersey 33, Neptune, NJ, United States

**Keywords:** indocyanine green, laparoscopic cholecystectomy, cholecystitis, percutaneous cholecystostomy

## Abstract

The use of indocyanine green for fluorescent cholangiography in patients with cholecystitis initially treated with percutaneous cholecystostomy drainage catheters was described in this two case series. Two patients underwent robotic assisted cholecystectomy with fluorescent cholangiography and indocyanine green through percutaneous cholecystostomy drainage catheters. The patients were diagnosed with acute cholecystitis. Directed injection of indocyanine green allowed for direct visualization of the biliary system allowing for a safe identification of the critical view of safety. Injection of indocyanine green for fluorescent cholangiography through percutaneous cholecystostomy drainage catheters is reliable to assess the critical view of safety and allows for improved identification of the biliary tree anatomy. Administration of indocyanine green through the percutaneous cholecystostomy drainage catheters avoided background hepatic fluorescence and increased contrast between biliary structures.

## Introduction

**Figure 1:**
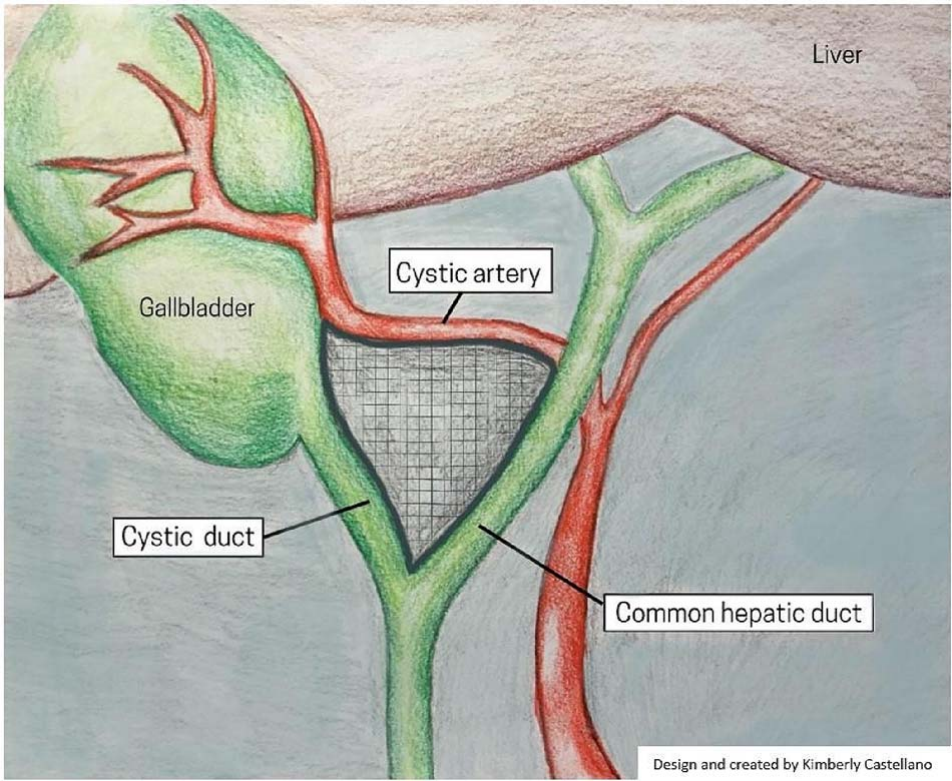
Illustrative depiction of Calot Triangle

The evolution of laparoscopic cholecystectomy (LC) since its implementation in 1988 has made it one of the most common abdominal surgical procedures today, with clear benefits for patients over open cholecystectomy, including reduced postoperative hospital stay, postoperative morbidity, postoperative wound infection, and time under anesthesia [1, 2]. However, the procedure has an increased risk of bile duct injury (BDI) (0.1%–1.5%) compared to open cholecystectomy (0.1%–0.2%) [3]. More recently, data suggests a decline of BDI (0.32%–0.52%) in LC without an increase in mortality or morbidity [4]. The reasons for BDI can be attributed to three general elements; surgical technique, the severity of disease, and abnormal surgical anatomy [5]. Within the last 5 years, studies have shown that a significant contributing factor to BDI is the misidentification of biliary anatomy (71%–97%) [6]. It is important to recognize that BDI and vasculobiliary injury (VBI) are preventable, and a large number of strategies have been explored to mitigate BDI and VBI [7]. The present review focuses on the critical view of safety (CVS) and intraoperative near-infrared fluorescent (NIRF) imaging using indocyanine green (ICG) strategies. CVS requires an in-depth understanding of Calot’s hepatobiliary anatomy and the essential steps of the technique; as demonstrated in Fig. 1. Emerging visualization techniques, such as ICG-NIRF, may enhance the effectiveness of CVS by providing real-time, intraoperative identification of the extrahepatic biliary system.

ICG is a safe, cost-effective, and widely available fluorescent dye traditionally administered intravenously (IV), where it is metabolized and excreted into the biliary system [8]. A significant drawback to ICG-NIRF imaging is the background fluorescence of hepatic structures [9]. Previous literature has explored the timing interval between IV administration and anesthesia induction ranging from 15 min to 24 h [9–12]. Further evolution of ICG visualization techniques that circumvent intrahepatic structures altogether, such as directly injecting ICG through a mature percutaneous cholecystostomy drainage catheter (PCDC) or intraoperative percutaneous needle puncture of the gallbladder have suggested improved visualization of biliary anatomy. This technique provides higher contrast between hepatic and extrahepatic biliary structures on NIRF images [13]. The case series below demonstrates the advantages of intracystic injection of ICG intraoperatively through percutaneous cholecystostomy tube.

### Case 1

A 59-year-old male with a past medical history of cholangitis and atrial fibrillation was diagnosed with moderate acute cholecystitis. He presented to an out-of-country hospital with abdominal pain from fatty food ingestion, fever, nausea, vomiting, and jaundice. The diagnosis was confirmed using computed tomography (CT) with gallstones and gallbladder wall thickening. In the same hospital course, the patient developed a fever, elevated liver enzymes, and septic shock. He was transferred to the intensive care unit (ICU) and underwent emergent percutaneous cholecystostomy tube placement as he was unfit for surgery during acute septic shock episode. The patient improved after PCDC placement and discharged with drain and outpatient follow up. The patient re-presented to our facility 3 weeks later for a syncopal episode and new onset of right lower extremity pain. His laboratory results showed slight transaminitis with an aspartate transaminase (AST) and alanine transaminase (ALT) of 58 and 72 U/L, respectively and a peak white blood cell count of 17 900 U/L. He was assessed by the cardiology team who did not identify any acute cardiac events and patient’s overall performance status had improved since prior hospitalization and septic shock episode. He was optimized from a cardiac perspective and ultimately cleared for surgical intervention. Pre-operatively, the patient underwent a magnetic resonance cholangiopancreatogram (MRCP) to assess for biliary obstruction prior to laparoscopic cholecystectomy. The MRCP was negative for biliary tract obstruction. The patient was scheduled for an interval robot-assisted LC as an outpatient. Eight days later, he underwent a robot-assisted NIRF guided LC using ICG. The patient described in case 1 had an uncomplicated postoperative course and he was discharged on the same day of surgery.

### Case 2

A 72-year-old female with a past medical history of hypertension and rheumatic fever with a well-functioning mechanical mitral valve (placed in 2003) and left ventricular ejection fraction of 55%–60%, paroxysmal atrial fibrillation on coumadin, chronic obstructive pulmonary disease, obstructive sleep apnea, gastroesophageal reflux disease, and class 3 obesity. She presented with intermittent right upper quadrant (RUQ) and epigastric pain that was progressively worsening. Pain was associated with nausea, vomiting, and diarrhea. Her laboratory results demonstrated a white blood cell count of 13 600 U/L, and a peak AST and ALT of 60 and 43 U/L, respectively. She underwent a RUQ abdominal ultrasound with positive sonographic Murphy’s sign, cholelithiasis, and biliary sludge suggestive of acute cholecystitis. The patient underwent subsequent hepatobiliary iminodiacetic acid scan, findings consistent with cystic duct obstruction. Due to her comorbidities and concurrent anticoagulation with high risk for intraoperative bleeding, she was not amenable to emergent surgery at that time. Therefore, PCDC was placed by the interventional radiology team. The patient was scheduled for outpatient follow-up in 6 weeks and discharged. She presented 6 weeks later with similar abdominal pain and received a RUQ ultrasound showing multiple gallstones, biliary sludge, and diffuse gallbladder wall thickening. Shared decision making was utilized once able to obtain cardiology recommendations. Ultimately, the patient cleared for surgery and anticoagulation was able to be held appropriately. At that time, the patient elected to undergo NIRF-guided robot-assisted LC using ICG.

The patient described in case 2 experienced a complication in the post-operative course with right upper quadrant pain and leukocytosis. Her white blood cell count peaked on postoperative Day 3 at 17800 U/L, she was treated with intravenous piperacillin–tazobactam 3.375 mg, and a computed tomography scan of the abdomen and pelvis without contrast was conducted on postoperative day 4; no drainable intra-abdominal collections identified. By postoperative day 6, her temperature and white blood cell count normalized, and she was subsequently discharged. She followed up at three and five weeks postoperatively. At these visits, the patient reports complete resolution of post-operative right upper quadrant pain. She did not experience any further complications.

### Surgery

**Figure 2:**
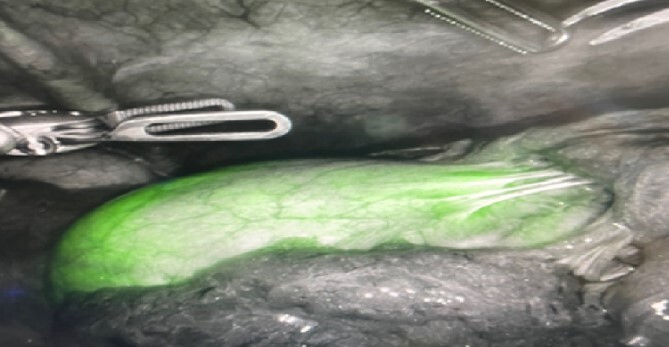
Initial illumination of gallbladder after ICG injection through PCDC.

**Figure 3:**
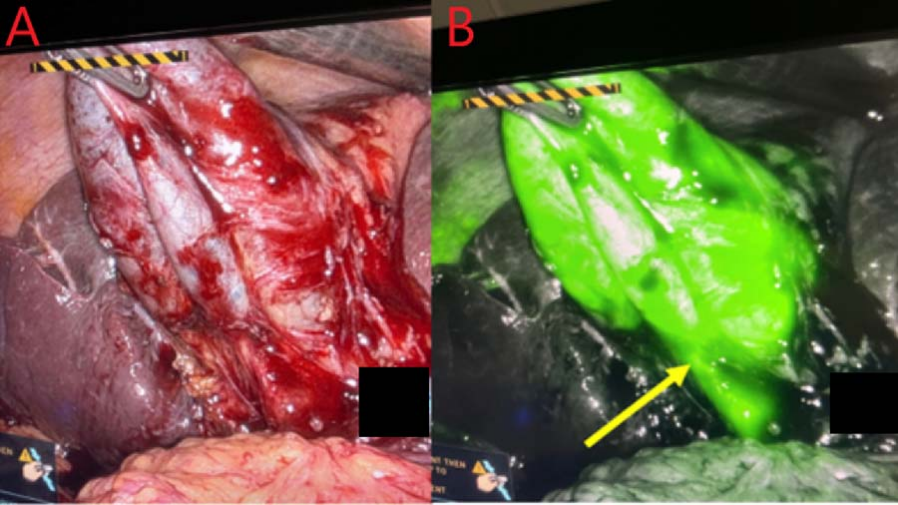
Gallbladder and cystic duct identification without (A) and with NIRF ICG (B). Arrow reflects improved cystic duct visualization.

**Figure 4:**
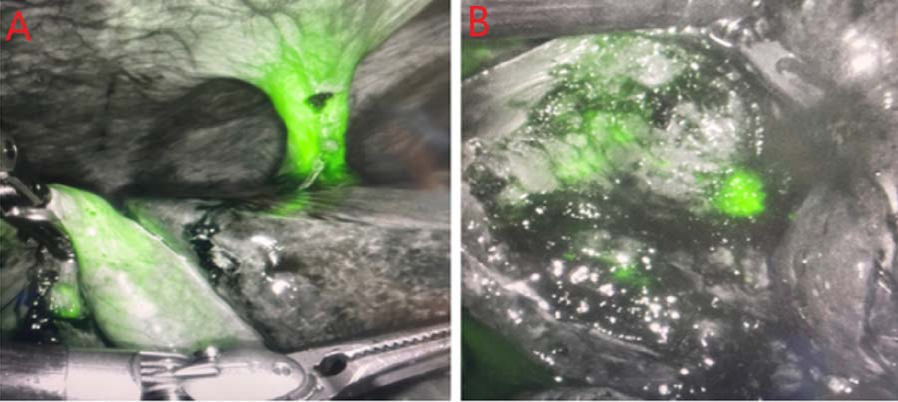
PCDC tract after administration of ICG. (A) is prior to surgical removal of gallbladder, (B) is postoperative liver bed.

In the peri-operative period, a bolus ICG solution (2.5 mg in 10 mL of sterile saline) was administered intracystically via percutaneous cholecystostomy drain within 1 hour of the operative start time. An infra-umbilical incision was made, and a Hasson trocar was placed and insufflated with 15 mmHg of carbon dioxide. A laparoscope was inserted to inspect the abdomen. An 8 mm 30-degree laparoscope was introduced, and three additional 8 mm trocars were placed, one in the epigastrium and two in the right upper quadrants. Laparoscopic near-infrared cholangiograms were captured using the Firefly system on the DaVinci robot, as demonstrated in Figs 2 and 3A and B and 4A) with Fig. 4B displaying the gallbladder fossa after removal.

In each case, the gallbladder was mobilized and the common bile and cystic ducts were visualized using Firefly. There was no extra biliary enhancement appreciated. Careful blunt dissection was conducted at the triangle of Calot until the cystic artery, cystic duct and gallbladder infundibular junction were identified, and CVS was obtained. At this point, the biliary anatomy was confirmed using the Firefly imaging. No additional NIRF enhancement was seen outside of the biliary anatomy, and the gallbladders were excised and removed using an EndoCatch bag through the umbilical trocar.

Robotic approach was performed at our institution for both cases based on surgeon preference and available equipment. This procedure could be performed using either robotic or standard laparoscopic technique depending on equipment available to the surgeon with ICF-NIRF capabilities.

## Discussion

ICG was approved by the FDA for clinical use in 1959 and was originally used as a quantitative analysis of hepatic and cardiac function [14]. Technological and imaging techniques limited the use of ICG in laparoscopic surgery until the early 2000s when digital advancements in imaging provided resolutions suitable for laparoscopic surgery [14]. Currently, ICG-NIRF is used in open, laparoscopic, and robotic cholecystectomies as a preoperative injection to aid in the visualization of biliary anatomy [15]. Following an injection of ICG, the compound rapidly binds to albumin and is exclusively excreted hepatically while emitting electromagnetic radiation at ~835 nm wavelength [16]. During an LC, infrared cameras, sensitive to this wavelength, can capture and generate an image visualizing the areas ICG is present [16, 17]. The advantages of this technique are the cost-effectiveness, availability, overall safety of the compound, and no additional need for further equipment [18]. Limitations of ICG may include limited spread of contrast in patients with biliary obstruction and leakage of ICG into the peritoneal cavity resulting in the loss of biliary enhancement [18].

In the present study, we proposed the intracystic injection of ICG through patent percutaneous cholecystostomy drains provided increased contrast of extra biliary structures from surrounding tissue, which may reduce the chances of iatrogenic biliary injury. Classically, ICG is introduced to the body via peripheral IV injection within 24 h of surgery. This strategy provides reliable hepatic excretion of ICG into the biliary system [15]. However, the hepatic and lymphatic tissue surrounding the gallbladder tends to fluoresce, reducing the contrast between the surgically important structures, namely the common bile duct and cystic duct [19]. In this case series, the biliary tree was visualized in 100% of cases where ICG was injected directly into the gallbladder compared to 83% using intravenous injection [19]. We used the intracystic injection of ICG to bypass hepatic structures in an attempt to reduce the background fluorescence and increase the contrast of biliary structures from surrounding tissue. Accurately and reliably identifying structures during the dissection and critical view of the gallbladder will reduce the risk of inadvertently injuring the biliary structures. Additionally, this technique may be beneficial in complex LC where extensive inflammation and adhesions require further dissection of the Calot triangle. Thus, in our experience, using the intracystic injection of ICG, we can perform LC with more confidence in routine and complex cases.

It is important to note, that the technique of ICG injection through PCDC would only be utilized when appropriate and pre-operative PCDC was indicated for management. Our case series does not suggest placing PCDC routinely to allow for intraoperative administration of ICG. In cases where PCDC is not indicated, surgeons may consider direct gallbladder indocyanine green injection technique with percutaneous injection as described by Cárdenas *et al.* [20]. Utilizing an already established and medically indicated PCDC, does not add any additional intraoperative time or equipment to the case and would likely minimizes the risk for intraabdominal ICG leakage as it is going through an already established PCDC tract. ICG through PCDC should be considered a relative alternative to ICG administration through IV and percutaneous gallbladder injection.

The sophistication of this technique is progressing. There is no standard for the timing and dosage of ICG administration in an LC. However, we can say that intravenous administration does not provide the level of contrast as an intracystic injection through a percutaneous cholecystostomy drain.

## Conclusion

The intracystic injection of ICG through patent percutaneous cholecystostomy drain enhances the contrast of the extrahepatic biliary anatomy from surrounding tissues compared to IV administration. The ability for accurate and reliable visualization of these structures throughout surgery may decrease the risk of iatrogenic injury to biliary structures and increase surgeon confidence in more complex LC.
